# Expanding Knowledge and Integration of Occupational Therapy in Vietnamese Healthcare: A Study of Educational Interventions and Cultural Relevance

**DOI:** 10.1155/oti/5812871

**Published:** 2025-03-14

**Authors:** John R. Patro, Jacqueline Daniel, Aiden Darcy

**Affiliations:** Department of Occupational Therapy, Monmouth University, West Long Branch, New Jersey, USA

## Abstract

Occupational therapy (OT) is an emerging healthcare profession in Vietnam, with limited awareness and understanding among local healthcare professionals (HCPs). This study is aimed at assessing the impact of an educational intervention designed to improve HCPs' knowledge and attitudes toward OT in Vietnamese healthcare settings. Utilizing a mixed-methods approach, the study involved 13 participants from Da Nang Orthopedics and Rehabilitation Hospital and Da Nang Psychiatric Hospital, representing various healthcare disciplines. The intervention included a series of journal club sessions focused on the principles, scope, and culturally relevant applications of OT. Quantitative data from pre- and postintervention assessments revealed significant improvements in HCPs' general knowledge of OT, its practice areas, and goals, with statistical significance observed in multiple domains. Qualitative data collected through field notes and reflective journals provided additional insights into HCPs' evolving perceptions of OT's role in holistic and mental healthcare. Notably, the study highlighted cultural factors, such as family involvement in patient care, which align with OT's client-centered approach but may also pose challenges in the integration of OT services. Barriers to OT adoption, including resource limitations and institutional support, were identified, underscoring the need for ongoing advocacy and resource allocation to sustain the progress achieved through the intervention. The findings suggest that culturally tailored educational programs are essential for the effective integration of OT in Vietnam and similar contexts. This research contributes to the global understanding of OT's role in diverse healthcare environments, providing a framework for expanding OT services in emerging regions.

## 1. Introduction

### 1.1. Overview of Occupational Therapy (OT) in Vietnam

OT is a rapidly emerging profession across Southeast Asia. However, in Vietnam, the field is still relatively new, with the first OT bachelor's programs established as recently as 2017. Despite this progress, there is a limited understanding of the role of OT among healthcare professionals (HCPs) in Vietnam. The lack of awareness and knowledge about OT's scope, methods, and impact has created challenges in integrating OT into multidisciplinary healthcare settings. As the healthcare system in Vietnam continues to develop, it is critical to educate HCPs on OT's contributions to patient care, particularly within mental health and rehabilitation settings [1, 2].

This manuscript presents a comprehensive study that investigates how educational interventions aimed at HCPs in Vietnam influence their understanding and attitudes toward OT. Specifically, this research evaluates the impact of an educational training program on the knowledge of OT among HCPs at two key institutions: Da Nang Psychiatric Hospital and Da Nang Orthopedics and Rehabilitation Hospital. Through a mixed-methods approach, this study explores the pre-existing knowledge gaps and measures the effect of tailored training sessions designed to improve the conceptualization of OT within these healthcare environments [3].

### 1.2. Problem Statement

The limited knowledge of OT among HCPs poses a significant barrier to the profession's advancement in Vietnam. Misunderstandings about the scope of OT's role in patient care lead to underutilization of OT services, which can hinder patient outcomes [4]. As Vietnam's healthcare system continues to expand, the need for culturally adapted OT services that align with local healthcare practices is more urgent than ever. This study addresses the knowledge gap through an educational intervention aimed at improving the understanding of OT's value and scope among HCPs in Vietnam [5].

### 1.3. Research Questions

This study seeks to answer the following research questions:

1. How does an educational training program influence HCPs' understanding of OT at Da Nang Orthopedics and Rehabilitation Hospital?
2. How does educational intervention affect the understanding of OT among orthopedic and psychiatric HCPs in Vietnam, and what cultural or systemic factors influence this understanding?

### 1.4. Theoretical Framework

The KAWA model serves as the theoretical foundation for this study, providing a culturally sensitive approach to understanding how HCPs in Vietnam conceptualize OT. Developed by Michael Iwama, the KAWA model uses the metaphor of a river to explain how individuals navigate their life's challenges, or “rocks,” within the broader environmental context, or “riverbanks” [6]. In this study, the KAWA model helps explore the cultural nuances that shape HCPs' understanding of OT and identifies the “rocks” (barriers) that inhibit the integration of OT into healthcare practice in Vietnam [7].

### 1.5. Significance of the Study

This study is among the first to evaluate the effectiveness of an OT-based educational intervention in Vietnam. By enhancing knowledge and attitudes toward OT, the findings will contribute to developing OT services that are better aligned with the needs of both HCPs and patients in Vietnam [8]. Additionally, the study contributes to global literature on the role of OT in emerging healthcare settings, providing valuable insights for the international expansion of OT services [9].

### 1.6. Section Outline

This manuscript is organized into five main sections.  Section 1 introduces the study, provides background information on OT in Vietnam, and outlines the research questions and theoretical framework.  Section 2 presents a comprehensive literature review, exploring previous research on OT's role in healthcare and highlighting the existing knowledge gaps.  Section 3 details the methods used in this study, including the educational intervention design, participant selection, and data collection process.  Section 4 presents the study's findings, including both qualitative and quantitative data analysis. Finally, Section 5 discusses the implications of the findings, offering recommendations for future OT research, education, and practice.

## 2. Literature Review

### 2.1. Introduction

OT is a profession that emphasizes holistic, client-centered approaches to care. Its core philosophy is grounded in the belief that engaging in meaningful activities promotes health and well-being [1]. Despite the well-documented benefits of OT across multiple healthcare domains, awareness and understanding of OT's role and scope remain limited in several regions of the world, including Southeast Asia. This literature review provides a comprehensive examination of the global and regional contexts of OT, with a specific focus on Vietnam. The review explores existing research on HCPs' knowledge of OT, the impact of educational interventions, and cultural factors affecting the integration of OT into healthcare systems.

### 2.2. OT in Global and Southeast Asian Contexts

The profession of OT has gained substantial recognition in Western countries, where it is widely integrated into healthcare systems. In countries like the United States, Canada, and the United Kingdom, OT practitioners are key members of interprofessional teams, contributing to the rehabilitation and well-being of individuals with physical, cognitive, and psychosocial impairments [3]. However, the scope and practice of OT vary significantly across regions. In Southeast Asia, OT is still considered a novel profession, and its integration into healthcare systems has been uneven [4].

Several Southeast Asian countries, including Malaysia, Thailand, and the Philippines, have made strides in incorporating OT into healthcare education and practice. For example, Malaysia introduced its first OT program in the early 1970s and has since expanded OT services in both public and private healthcare settings [5, 10, 11]. However, Vietnam lags behind its regional neighbors in terms of OT development. Despite the recent establishment of OT training programs, the profession is still in its infancy, and there is a pressing need for advocacy and education to raise awareness among HCPs and the public [11].

### 2.3. HCPs' Knowledge of OT

One of the major barriers to the development of OT in emerging regions is the lack of knowledge and awareness among HCPs. Research conducted in various countries has consistently shown that healthcare providers, including physicians, nurses, and physical therapists, have limited understanding of OT's scope of practice [7, 8]. This gap in knowledge not only hinders interprofessional collaboration but also limits the referral of patients to OT services, ultimately impacting patient outcomes [9].

A study by Darawsheh in Jordan revealed that nearly half of the surveyed HCPs had little to no knowledge of OT, and many held misconceptions about the profession [12]. Similarly, research from Nigeria found that healthcare providers were unclear about OT's distinct role from other allied health professions [13]. These findings underscore the importance of targeted educational interventions to improve knowledge and understanding of OT among healthcare workers in settings where the profession is not well established [14].

### 2.4. OT in Vietnam

In Vietnam, the concept of OT is relatively new. The first OT training programs were launched in 2017, with the support of international organizations like the Medical Committee Netherlands–Vietnam (MCNV) [1, 2]. Despite these efforts, the profession remains underdeveloped, and there are few qualified OTs practicing in the country. HCPs in Vietnam, particularly in rural areas, often have limited exposure to OT, which can lead to misunderstandings about its role and benefits [15].

A 2022 study highlighted the need for educational interventions to improve HCPs' understanding of OT in Southeast Asia. The authors argued that educational initiatives should not only focus on disseminating knowledge about OT's scope but also emphasize its unique contributions to patient care, particularly in holistic and client-centered approaches [16].

### 2.5. The Role of Educational Interventions

Educational interventions have been shown to be effective in improving HCPs' knowledge of OT. Research has demonstrated that structured educational programs, such as workshops, seminars, and journal clubs, can significantly enhance participants' understanding of OT's role and scope [17]. For example, a study in Saudi Arabia found that an educational intervention targeting key healthcare professionals led to a potential increase in referrals to OT services due to educational efforts [18]. This further supports the premise that targeted education could foster enhanced collaboration between OTs and other HCPs [19].

In Vietnam, where OT is still emerging, educational interventions are particularly crucial. Introducing HCPs to the core principles of OT and its applications in different healthcare settings can promote greater utilization of OT services, ultimately improving patient outcomes [10]. Furthermore, educational interventions that are culturally sensitive and aligned with local healthcare practices, such as the use of the KAWA model, can be particularly effective in bridging knowledge gaps [7, 20].

### 2.6. Cultural Considerations in OT Education

Cultural factors play a significant role in shaping HCPs' understanding and acceptance of new practices, including OT. The KAWA model, developed by Michael Iwama, emphasizes the importance of considering cultural context in OT practice [7, 20, 21]. In many non-Western countries, HCPs may have different perceptions of health, illness, and rehabilitation, which can influence how they understand the role of OT. For example, in South Asian culture, family plays a central role in caregiving, and traditional approaches to rehabilitation often emphasize physical recovery over psychosocial well-being [22].

Educational interventions in Vietnam must therefore be designed with these cultural considerations in mind. The KAWA model, which uses the metaphor of a river to explore how individuals navigate life's challenges, is particularly useful in this context because it allows HCPs to reflect on their own cultural beliefs and how they intersect with OT principles [20]. By incorporating culturally relevant frameworks into OT education, it is possible to foster a deeper understanding and acceptance of the profession [23].

### 2.7. Gaps in the Literature

While there is growing literature on the global development of OT, there remains a significant gap in research specifically focused on Southeast Asia, particularly Vietnam. Few studies have explored the impact of educational interventions on HCPs' knowledge of OT in emerging regions, and even fewer have examined how cultural factors influence the integration of OT into healthcare systems [24]. This study is aimed at addressing these gaps by providing empirical data on the effectiveness of an educational intervention in improving knowledge of OT among HCPs in Vietnam.

### 2.8. Summary

The literature reviewed highlights the challenges and opportunities associated with introducing OT in emerging regions, particularly in Vietnam. HCPs' limited knowledge of OT is a significant barrier to the profession's growth, and educational interventions are necessary to address these gaps. This study builds on existing research by exploring the impact of a culturally sensitive educational program on HCPs' understanding and attitudes toward OT in two major healthcare institutions in Vietnam. By addressing both the knowledge gap and cultural considerations, this research is aimed at contributing to the sustainable development of OT in Vietnam and other emerging regions.

## 3. Methods

### 3.1. Introduction

This section details the research design, methodology, and procedures used to conduct this study. The goal of this research is to assess the impact of an educational training program on HCPs' knowledge and attitudes toward OT in Vietnam. A mixed-methods case study design was employed to gather both quantitative and qualitative data from participants at two institutions: Da Nang Orthopedics and Rehabilitation Hospital and Da Nang Psychiatric Hospital.

### 3.2. Research Design

A mixed-methods approach was chosen for this study to capture both the measurable outcomes of the educational intervention (quantitative data) and the more nuanced perceptions and experiences of the HCPs (qualitative data). By combining these approaches, the study provides a comprehensive understanding of the effect of the training program on the participants' knowledge and attitudes toward OT.

### 3.3. Project Planning and Development

The project was planned in collaboration with administrators and department heads at Da Nang Orthopedics and Rehabilitation Hospital and Da Nang Psychiatric Hospital. The first phase of planning involved a needs assessment to determine the existing level of knowledge of OT among HCPs at these sites. Following the needs assessment, the educational training program was developed, with journal club sessions serving as the primary format for delivering the educational content.

The development of the training program was guided by both Western OT principles and culturally sensitive approaches, including the KAWA model, to ensure the material was relevant and aligned with local healthcare practices. The program included three journal club sessions held over the course of 3 weeks, with each session focusing on different aspects of OT practice, such as task-oriented training, functional mobility, and client-centered care.

### 3.4. Participants

The participants for this study were recruited from Da Nang Orthopedics and Rehabilitation Hospital and Da Nang Psychiatric Hospital. Participants were recruited using convenience sampling through collaboration with department leaders at Da Nang Orthopedics and Rehabilitation Hospital and Da Nang Psychiatric Hospital. Recruitment materials, including informational flyers, were distributed within hospital departments, and in-person communication was utilized to engage HCPs. Eligibility criteria required participants to be employed at the respective hospitals, possess internet access for completing surveys, and commit to attending all scheduled educational sessions. This approach ensured a diverse, interprofessional sample, including physical therapists, physicians, and speech pathologists, to enhance the breadth of perspectives and focus on improving understanding of OT through targeted training programs.

The sample consisted of 20 HCPs from various disciplines, including physical therapy, medicine, nursing, and speech therapy. The sample size was determined based on the availability and accessibility of HCPs at Da Nang Orthopedics and Rehabilitation Hospital and Da Nang Psychiatric Hospital, employing a convenience sampling approach. Recruitment was guided by the capacity of the educational sessions, ensuring that participants could engage meaningfully with the content and that data collection would yield robust, analyzable results. A target sample size was not predetermined; instead, participation was maximized by distributing informational flyers, leveraging department leaders' networks, and ensuring broad representation across disciplines, such as physical therapy, medicine, and speech pathology. The final sample size was shaped by the number of eligible HCPs who met the inclusion criteria, provided informed consent, and committed to completing the pre- and postintervention components of the study. This flexible approach ensured the feasibility of the study while maintaining sufficient participant diversity to draw meaningful insights.

Thirteen participants of the 20 recruited were eligible for inclusion in the data analysis, having met the following inclusion criteria:

1. Employed as a licensed HCP in Vietnam
2. Available to attend all three journal club sessions
3. Willing to participate in pre- and posttraining assessments

Exclusion criteria included the following:

1. Individuals unable to provide consent or who refused to sign the electronic consent form
2. Those unable to complete the full educational program

The project was designed to include all HCPs, such as physical therapists, medical physicians, nurses, social workers, psychologists, doctors, and public health staff members.

### 3.5. Ethical Considerations

This approved study was conducted in accordance with Monmouth University's Human Research Protection Program and Institutional Review Board (IRB) guidelines (Approval #FA2311; December 22, 2023). Participants were informed of the study's purpose, procedures, and voluntary nature. Informed consent was obtained electronically through Google Forms, and all participants were assured of their anonymity and the confidentiality of their responses. Participants were informed that they could withdraw from the study at any time without any consequences. The research protocol ensured that the educational program was available to all HCPs at the two institutions, regardless of their participation in the study.

### 3.6. Instrumentation

To measure changes in participants' knowledge and attitudes toward OT, a 68-item questionnaire was adapted from a validated instrument developed by Abu Tariah et al. [5]. Permission to use the questionnaire and translate it into Vietnamese was obtained directly from the questionnaire's author via electronic communication. A trusted translator, familiar with the study, conducted a verbatim translation of the published questionnaire into Vietnamese. The translated version was subsequently reviewed and approved by the Da Nang Psychiatric Hospital administrator and the Chief Physical Therapist at the Da Nang Orthopedic Hospital. Both reviewers confirmed the translation's accuracy, and no edits were required.

The questionnaire was divided into five categories:

1. General knowledge about OT
2. Knowledge about areas of OT practice
3. Knowledge about goals of OT practice
4. Knowledge about OT treatment methods and media
5. General impressions of OT

The questionnaire used a 5-point Likert scale (ranging from “*strongly agree*” to “*strongly disagree*”) to measure participants' responses. In addition to the questionnaire, participants' reflections on the educational program were collected through guided journaling and field notes recorded by the student investigator.

### 3.7. Data Collection

Data were collected before and after the educational intervention. Prior to the first journal club session, participants completed the pretest questionnaire to assess their baseline knowledge of OT. Following the final session, participants completed the posttest questionnaire to determine changes in their knowledge and attitudes toward OT.

The qualitative data were collected through field notes and reflective journaling. The student investigator maintained detailed field notes during each session, recording observations of participant engagement, discussions, and informal feedback. Participants were also asked to maintain a reflective journal, where they recorded their thoughts and insights following each session. These journals were collected and analyzed for thematic content.

### 3.8. Procedures

The educational intervention consisted of three journal club sessions, each lasting approximately 1 h. The journal club sessions were led by two entry-level OT doctoral candidates who were completing their doctoral capstone projects at the identified sites. These candidates are the second and third authors of this study. Both candidates had completed all required didactic coursework and fieldwork for their OT doctoral program, demonstrating entry-level competence to facilitate the sessions effectively.

The candidates were supervised by the study's primary author, who provided oversight and guidance throughout the intervention. Before facilitating the sessions, they consulted with the primary author to collaboratively determine the content of each journal club and developed all associated materials for institutional approval. Their preparation and expertise ensured the sessions were aligned with the study's objectives and met the participants' needs.

This combination of academic training, fieldwork experience, and collaboration with the supervising author positioned the doctoral candidates to successfully lead the sessions, incorporating interactive learning methods such as presentations, group discussions, and case studies.

The sessions were designed to be interactive, incorporating presentations, group discussions, and case studies. The content of each session was tailored to the specific needs of the participants, based on the results of the needs assessment conducted during the planning phase.

• Session 1: focused on introducing OT and its scope of practice. The session provided an overview of the role of OT in rehabilitation and patient care, with an emphasis on task-oriented training and functional mobility.
• Session 2: focused on OT interventions in psychiatric care, highlighting the importance of client-centered care and therapeutic activities in mental health settings.
• Session 3: focused on integrating OT into interprofessional teams. This session emphasized the value of OT in collaborative care settings and explored strategies for improving referrals and collaboration between OTs and other HCPs.

### 3.9. Data Analysis

#### 3.9.1. Quantitative Data Analysis

The quantitative data from the pre- and posttest questionnaires were analyzed using descriptive statistics and paired *t*-tests to determine whether there were significant differences in the participants' knowledge and attitudes before and after the intervention. The analysis focused on comparing the mean scores for each category (e.g., general knowledge, areas of OT practice) to assess the overall impact of the training program.

#### 3.9.2. Qualitative Data Analysis

The qualitative data from the field notes and reflective journals were analyzed using a thematic analysis approach. The data were coded based on recurring themes, such as participant perceptions of OT, cultural barriers, and interprofessional collaboration. Thematic analysis allowed for the identification of key insights and experiences that could not be captured through quantitative methods alone.

### 3.10. Trustworthiness

To ensure the trustworthiness of the qualitative findings, multiple strategies were employed:

• Triangulation: Data were collected from multiple sources, including pre- and posttest questionnaires, field notes, and reflective journals, to ensure the consistency of the findings.
• Member checking: Participants were given the opportunity to review and provide feedback on the preliminary findings to ensure accuracy and authenticity.
• Peer review: The research design, data collection, and analysis processes were reviewed by faculty mentors to ensure methodological rigor.

### 3.11. Summary

This section outlined the research design, methodology, and procedures used to investigate the impact of an educational intervention on HCPs' knowledge and attitudes toward OT in Vietnam. The study employed a mixed-methods approach, combining quantitative and qualitative data to provide a comprehensive analysis of the training program's effectiveness. The next section will present the findings from the data analysis.

## 4. Findings

### 4.1. Introduction

This section presents the findings from both the quantitative and qualitative data collected during the study. The purpose of this study was to assess how an educational training program impacted HCPs' knowledge and attitudes toward OT at Da Nang Orthopedics and Rehabilitation Hospital and Da Nang Psychiatric Hospital. The results are organized according to the research questions, focusing first on the quantitative analysis of pre- and posttest questionnaire data, followed by the thematic analysis of qualitative data from field notes and reflective journals.

### 4.2. Quantitative Findings

#### 4.2.1. Participant Demographics

Thirteen HCPs (*n* = 13) participated in both the pre- and posttraining assessments. The participants represented a variety of disciplines, including physical therapy, medicine, speech therapy, and nursing.  Table 1 provides an overview of the demographic characteristics of the participants.

#### 4.2.2. General Knowledge About OT

The participants' general knowledge of OT was assessed through a set of 10 items in both the pre- and posttest questionnaires. The results indicated a significant improvement in general knowledge following the educational intervention.  Table 2 provides a summary of the pre- and posttest scores for general knowledge of OT.

As shown in Table 2, the mean score for general knowledge of OT increased from 2.80 in the pretest to 4.30 in the posttest, indicating a statistically significant improvement in knowledge (*p* < 0.001).

#### 4.2.3. Knowledge About Areas of OT Practice

Participants were asked to rate their knowledge of different areas of OT practice, including rehabilitation, mental health, and pediatrics.  Table 3 shows the changes in knowledge about OT practice areas.

The data reveal that participants showed significant improvements in their understanding of OT across all practice areas. The largest gains were seen in the mental health and pediatric domains, where knowledge levels increased substantially.

#### 4.2.4. Knowledge About Goals of OT Practice

Participants were also assessed on their knowledge of the goals of OT, including the promotion of independence, client-centered care, and functional recovery. The posttest results indicated a considerable improvement in this domain, as shown in Table 4.

The data suggest that participants' understanding of OT goals improved significantly following the educational intervention. There was a strong increase in awareness of OT's focus on promoting independence and facilitating client-centered care.

### 4.3. Qualitative Findings

#### 4.3.1. Thematic Analysis

The qualitative data collected from field notes and reflective journals were analyzed using thematic analysis. Several key themes emerged from the data, reflecting participants' perceptions of the educational intervention, their evolving understanding of OT, and the cultural factors influencing their views on rehabilitation and patient care.

##### 4.3.1.1. Theme 1: Increased Awareness of OT's Role

Participants reported a significant increase in their understanding of OT's role within healthcare teams. Prior to the intervention, many participants viewed OT as a supplementary service focused primarily on physical rehabilitation. However, following the journal club sessions, participants expressed a broader understanding of OT's role, including its contributions to mental health and client-centered care.

One participant noted:

I always thought OT was just for physical problems, but now I see how important it is in mental health too. It's about helping the whole person.

##### 4.3.1.2. Theme 2: Cultural Relevance of OT

Participants reflected on how the concept of OT aligned with their cultural views on caregiving and rehabilitation. Several participants highlighted the importance of family involvement in patient care, which is a common cultural practice in Vietnam. Participants discussed how OT's emphasis on client-centered care could be integrated with traditional caregiving practices to enhance rehabilitation outcomes.

One participant remarked:

In Vietnam, the family plays a big role in care. OT's approach of including family in treatment makes sense for us. It respects our culture.

##### 4.3.1.3. Theme 3: Barriers to OT Integration

Despite the positive reception of OT concepts, participants identified several barriers to integrating OT into their practice. These included a lack of resources, limited staff trained in OT, and competing demands from other disciplines such as physical therapy and nursing. Some participants expressed concerns about how to advocate for OT in their institutions, given the scarcity of OTs in Vietnam.

One participant commented:

We understand now that OT is valuable, but we don't have the resources or enough staff. It's hard to make the case for OT when we're already short on personnel.

### 4.4. Cross-Case Findings

A cross-case analysis of the two study sites (Da Nang Orthopedics and Rehabilitation Hospital and Da Nang Psychiatric Hospital) was conducted to compare the impact of the educational intervention across different healthcare settings. The analysis revealed that participants from both sites showed similar improvements in knowledge and attitudes, but there were notable differences in how OT was perceived depending on the healthcare context.

At Da Nang Psychiatric Hospital, participants were particularly receptive to OT's role in mental healthcare, whereas participants at Da Nang Orthopedics and Rehabilitation Hospital focused more on OT's role in physical rehabilitation. These differences highlight the need for tailored educational programs that address the specific needs and contexts of different healthcare institutions.

### 4.5. Summary

The findings from this study demonstrate that the educational training program had a significant positive impact on HCPs' knowledge and attitudes toward OT in Vietnam. Both quantitative and qualitative data indicate that participants gained a deeper understanding of OT's scope, goals, and contributions to healthcare. However, challenges remain in terms of integrating OT into practice, particularly in resource-limited settings. The next section will discuss these findings in greater detail and explore their implications for OT education, practice, and future research.

## 5. Discussion

### 5.1. Introduction

This section discusses the implications of the study's findings, with a focus on how the educational intervention improved HCPs' knowledge and attitudes toward OT. It also examines the challenges identified in integrating OT into healthcare practice in Vietnam, including cultural and systemic barriers, and provides recommendations for future research and practice.

### 5.2. Key Findings and Their Implications

#### 5.2.1. Improved Knowledge and Attitudes Toward OT

The study demonstrated that the educational intervention had a significant impact on HCPs' knowledge and attitudes toward OT. Participants showed marked improvements in their understanding of OT's role, practice areas, and goals. This finding aligns with previous research indicating that targeted educational interventions can be effective in improving HCPs' knowledge of OT in settings where the profession is underdeveloped [17, 18].

The increase in knowledge about OT's contributions to mental healthcare is particularly noteworthy, as this area had previously been underappreciated by many participants. This shift in perception is critical, given the growing recognition of the importance of mental health in overall well-being. By broadening HCPs' understanding of OT's role beyond physical rehabilitation, the intervention has the potential to promote more holistic approaches to patient care [21].

#### 5.2.2. Barriers to OT Integration

While the educational intervention succeeded in improving knowledge, the qualitative findings revealed several barriers to integrating OT into everyday practice. Resource limitations, including the shortage of trained OTs, were frequently cited as challenges. Participants expressed concerns about how to implement OT services without sufficient staff or institutional support. These barriers are consistent with findings from other studies in resource-limited settings, where the integration of OT is often hampered by competing demands and a lack of resources [24].

The cultural context also plays a critical role in the acceptance and integration of OT. In Vietnam, family involvement in caregiving is deeply embedded in healthcare practices. While this cultural norm aligns well with OT's client-centered approach, it also presents challenges, as family members may have differing expectations about rehabilitation outcomes and care strategies [22]. Future OT interventions in Vietnam will need to consider these cultural factors and work closely with families to ensure that OT services are fully integrated into patient care.

### 5.3. Recommendations for OT Practice and Education

The findings from this study highlight several key recommendations for advancing OT practice and education in Vietnam:

1. Develop culturally tailored OT programs: Given the cultural importance of family involvement in patient care, OT programs should be designed to include family members in the rehabilitation process. This approach will help bridge the gap between traditional caregiving practices and modern OT interventions [23].
2. Increase advocacy for OT resources: There is a clear need for increased advocacy to secure resources for OT services, including hiring more trained OTs and providing continuing education for existing staff. HCPs who participated in this study are now better equipped to advocate for the inclusion of OT in their institutions, but they will need support from policymakers and administrators to make these changes sustainable.
3. Expand mental health OT services: The positive reception of OT's role in mental healthcare suggests that there is potential for expanding OT services in psychiatric settings. Future educational programs should continue to emphasize OT's contributions to mental health and explore ways to integrate OT into existing mental health services in Vietnam [20].
4. Provide continuing education opportunities: Given the limited exposure to OT in Vietnam, continuing education programs are essential for keeping HCPs informed about the latest developments in OT practice. Journal clubs, workshops, and interprofessional training sessions should be regularly offered to help maintain and build upon the knowledge gained through this intervention [18].

### 5.4. Limitations of the Study

While this study provides valuable insights into the impact of educational interventions on HCPs' knowledge and attitudes toward OT, there are several limitations that should be acknowledged:

• Sample size: The sample size of 13 participants limits the generalizability of the findings. Future research should be aimed at including a larger sample size to provide more robust conclusions.
• Resource limitations: The study was conducted in resource-limited settings where the availability of OT services and staff is constrained. These limitations may have influenced participants' perceptions of OT and their ability to integrate OT into practice.

### 5.5. Future Research Directions

Building on the findings from this study, future research should focus on the following:

• Longitudinal studies: conducting longitudinal studies to assess the long-term impact of educational interventions on HCPs' knowledge and practice of OT
• Interprofessional collaboration: exploring how OT can be more effectively integrated into interprofessional healthcare teams in Vietnam, particularly in resource-limited settings
• Cultural adaptation of OT models: investigating how existing OT models, such as the KAWA model, can be further adapted to fit the cultural context of Vietnam and other Southeast Asian countries [6, 7, 20, 21]

### 5.6. Conclusion

The educational intervention demonstrated significant improvements in HCPs' knowledge and attitudes toward OT in Vietnam. By broadening participants' understanding of OT's role and practice areas, particularly in mental health, the intervention has the potential to promote more holistic approaches to patient care. However, the successful integration of OT into healthcare systems in Vietnam will require ongoing advocacy, resource allocation, and culturally tailored approaches to education and practice. This study provides a foundation for future efforts to expand and enhance OT services in Vietnam and other emerging healthcare settings.

## Figures and Tables

**Table 1:** Participant demographics.

**Discipline**	**Level of education**	**Years of training**	**Number of participants (%)**
Physical therapy	Bachelor's	4 years	8 (62%)
Medicine	Master's	6 years	2 (15%)
Speech therapy	Bachelor's	3 years	2 (15%)
Nursing	Bachelor's	3 years	1 (8%)

**Table 2:** General knowledge of occupational therapy (pre- and posttest scores).

**Knowledge area**	**Pretest mean (SD)**	**Posttest mean (SD)**	**p** ** value (** **t** ** -test)**
General knowledge of OT	2.80 (0.62)	4.30 (0.50)	< 0.001

**Table 3:** Knowledge about areas of occupational therapy practice (pre- and posttest scores).

**Area of OT practice**	**Pretest mean (SD)**	**Posttest mean (SD)**	**p** ** value (** **t** ** -test)**
Rehabilitation	2.92 (0.70)	4.15 (0.60)	< 0.001
Mental health	2.50 (0.80)	4.00 (0.65)	< 0.001
Pediatrics	2.30 (0.82)	3.95 (0.70)	< 0.001

**Table 4:** Knowledge about goals of occupational therapy practice (pre- and posttest scores).

**OT goal**	**Pretest mean (SD)**	**Posttest mean (SD)**	**p** ** value (** **t** ** -test)**
Promote independence	2.75 (0.65)	4.25 (0.55)	< 0.001
Client-centered care	2.65 (0.72)	4.10 (0.60)	< 0.001
Functional recovery	2.80 (0.68)	4.35 (0.50)	< 0.001

## Data Availability

The data that support the findings of this study are available on request from the corresponding author. The data are not publicly available due to privacy or ethical restrictions.
